# Drug-induced lung disease adverse effect with Ledipasvir Acetonate/Sofosbuvir

**DOI:** 10.1186/s40780-020-00162-y

**Published:** 2020-04-02

**Authors:** Sachiko Omotani, Toshihiko Ishizaka, Miki Inoue, Koji Nishida, Yukako Yasui, Yasutoshi Hatsuda, Junji Mukai, Michiaki Myotoku

**Affiliations:** 1 Laboratory of Practical Pharmacy and Pharmaceutical Care, Faculty of Pharmacy, Osaka Ohtani University, 3-11-1, Nishikiori-kita, Tondabayashi, Osaka, 584-8540 Japan; 2 Sakai City Medical Center, 1-1-1 Ebaraji-cho, Nishi-ku, Sakai, Osaka, 593-8304 Japan

**Keywords:** Chronic hepatitis C, Direct acting antivirals (DAAs), Interstitial lung disease, Drug-induced lung disease

## Abstract

**Background:**

Interferon and ribavirin have been used as therapeutic agents for chronic hepatitis C infection or C-compensated cirrhosis in the conventional treatment. Hepatitis C virus (HCV) -specific direct-acting antiviral agents that directly inhibit the growth process of HCV have been approved since 2011. However, in the early post-marketing vigilance phase of ledipasvir acetonate/sofosbuvir (LDV/SOF), there were reports of interstitial lung disease in 4 out of 32,700 cases with death in 1 case; the onset mechanism is unknown.

**Case presentation:**

Treatment for hepatitis C was deemed to be necessary, and the patient was referred to our hospital. Oral administration of LDV/SOF was started. On day 8 of administration, a fever of 38–39 °C and coughing were observed followed by the gradual appearance of shortness of breath. As there was no improvement, the patient visited her primary care physician on day 16 of administration and the patient was brought urgently to our hospital on the same day. Blood tests and imaging tests were conducted at our hospital on the day of emergency transport; inflammatory response markers showed abnormal values, and sialylated carbohydrate antigen Krebs von den Lungen-6 was within the normal value range at 303 U/mL. Because the possibility of infection was low based on results of imaging and bronchoalveolar lavage, drug-induced lung disease was suspected, LDV/SOF administration was discontinued, and steroid administration was started. Following steroid pulse therapy, treatment with oral prednisolone tablets was gradually tapered. The patient’s symptoms were relieved and she was discharged.

**Conclusions:**

The patient’s medication history in this case indicated that there were no drugs taken before or after administration of LDV/SOF until the adverse reaction occurred, and there were no supplements or dietary supplements taken. Therefore, LDV/SOF has been proposed as the cause of the suspected adverse effect. Pharmacists should try to collect adverse effect reports to identify adverse effects early.

## Background

Interferon (IFN) and ribavirin have been used as the standard treatment of chronic hepatitis C infection and hepatitis C-compensated cirrhosis. The first hepatitis C virus (HCV)-specific direct-acting antiviral agents (DAAs) that directly inhibit the growth process of HCV were approved in 2011. DAAs were initially administered in combination with IFN, but in 2014 an IFN-free DAA treatment was approved. HARVONI Combination Tablet (ledipasvir acetonate/sofosbuvir [LDV/SOF]; Gilead Science Inc., Tokyo, Japan) is a drug with efficacy and an effect of “amelioration of viremia in serogroup 1 (genotype 1) chronic hepatitis C or type C-compensated cirrhosis.” This drug was approved for the treatment of genotype 1 chronic hepatitis C infection in the United States in 2014 and in Japan in 2015. Approval of LDV/SOF for additional indication for “amelioration of viremia in serogroup 2 (genotype 2) chronic hepatitis C or type C-compensated cirrhosis” was obtained in February 2018. It has been reported that administration of LDV/SOF to patients with genotype 1 chronic hepatitis C, including those with compensated cirrhosis, resulted in 100% of the patients achieving a sustained virologic response after 12 weeks [1, 2].

In the 7th edition of the HCV treatment guidelines [3], DAAs are mentioned as first-choice drugs that are essential for the treatment of hepatitis C. Although treatment with IFN has shown adverse effects in almost all patients, treatment with DAAs has few adverse effects and the response to treatment is also good as indicated above. DAAs have been widely used in recent years. The incidence of adverse reactions after a single administration of LDV/SOF was 19.1% according to the results of a phase 3 trial in Japan [1, 2], and the breakdown was headache (3.1%) as the most common, followed by nausea and constipation/pruritus (2.4% each), and sores (1.7%). The incidence of interstitial pneumonia as an adverse effect is between ≥0.1 and < 5% in the case of Interferon Alfa (NAMALWA) [4] according to the package insert, and there have been reports for other IFNs. The only cases of interstitial lung disease as an adverse effect of DAAs were the spontaneous report of simeprevir sodium [5] used in combination with IFN and the simultaneous administration of daclatasvir hydrochloride and asunaprevir [6]. LDV/SOF does not specify interstitial lung disease as an adverse effect in its package insert [7]. In the early post-marketing phase vigilance [8] of LDV/SOF, there were reports of interstitial lung disease in 4 out of 32,700 cases with death in 1 case; however, the onset mechanism of this adverse effect remains unknown. Here we report a case of hepatitis C in which LDV/SOF administration caused drug-induced lung disease.

## Case presentation

**Fig. 1:**
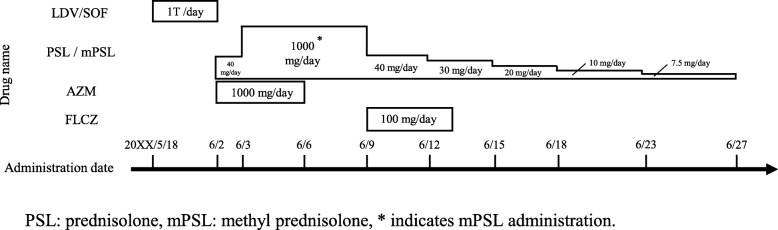
Medications taken

The main blood test results and dosing status are shown in Table 1 and Fig. 1. A 55-year-old woman, who was 158.0 cm tall and weighed 46.6 kg, had been treated for rheumatoid arthritis for one year until 6 years previously. There was no history of drug allergy or side effects. A positive HCV test with high titer was found at a medical examination 5 years previously, and the patient was referred to Sakai City Medical Center.

**Table 1:** Results of blood tests

Blood collection date (mm/dd)		3/24	6/2	6/3	6/4	6/6	6/9	6/22	7/6	9/13
WBC	(/μL)	4740	**10,100**	**13,450**	**10,170**	**10,090**	4410	4950	4940	4770
Neutro	(%)	56.0	**79.8**	**88.7**	**85.8**	**83.0**	56.2	79.1	45.3	43.4
AST	(U/L)	30	**69**	**68**	**52**	**49**	**55**	22	19	16
ALT	(U/L)	**43**	**68**	**109**	**103**	**93**	**109**	30	15	12
LDH	(U/L)	170	**941**	**1134**	**916**	**550**	**411**	224	175	160
CK	(U/L)	55	**384**	**501**	**388**	**203**	81	**35**	**32**	57
BUN	(mg/dL)	12.9	**21.3**	18.6	13.4	9.5	8.0	12.5	12.7	13.9
Cr	(mg/dL)	0.51	0.62	0.49	0.40	0.40	0.46	0.47	0.51	0.49
CRP	(mg/dL)	0.05	**4.20**	**5.45**	**2.52**	**0.69**	**0.35**	0.05	0.04	0.02
KL-6	(U/mL)		303							

Bold values indicate abnormal values

*WBC* white blood cell, *Neutro* neutrophils, *AST* aspartate aminotransaminase, *ALT* alanine aminotransferase, *LDH* lactate dehydrogenase,

*CK* creatine kinase, *BUN* blood urea nitrogen, *Cr* serum creatinine, *CRP* C-reactive protein, *KL-6* sialylated carbohydrate antigen Krebs von den Lungen-6

Examination revealed high HCV type 1 virus but normal liver function, so the patient was considered as a HCV carrier with persistently normal ALT and was followed-up.

**Fig. 2:**
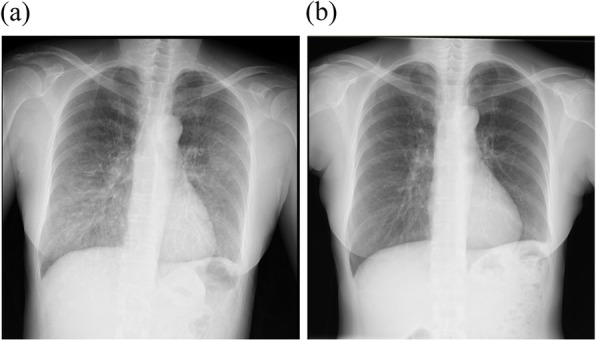
Chest roentgenogram findings. Each image has ground-glass opacity in the lower lung fields on both sides. **a** Image taken on June 2, 20XX, in the standing position, tube voltage of 130 kV, and tube current of 250 mA. **b** Image taken on June 22, 20XX, in the standing position, tube voltage of 130 kV, and current of 250 mA

**Fig. 3:**
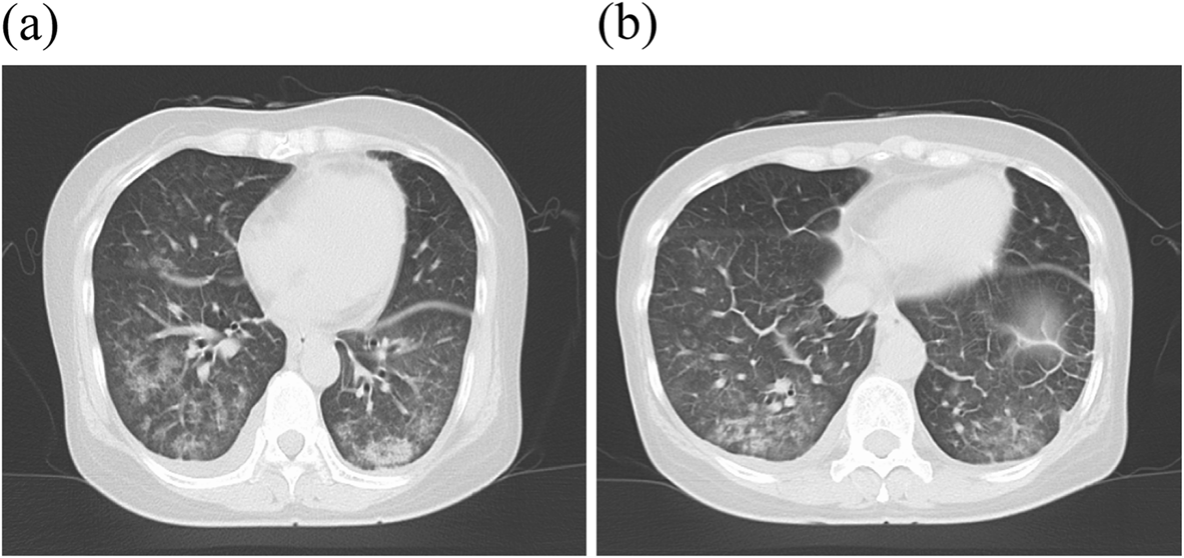
Computed tomography findings sliced at 5.0 mm intervals on June 2, 20XX. Both images have ground-glass opacity in the lower lung fields on both sides. **a** Image obtained at tube voltage of 120 kV and tube current of 107 mA. **b** Image obtained at tube voltage of 120 kV and tube current of 153 mA

Subsequent biochemical test results showed HCV positivity, slightly increased ALT of 54 (U/L), and increased HCV-RNA of 5.6 (Log IU/mL). Treatment for hepatitis C was deemed to be necessary, and the patient was referred to the hospital. Oral administration of LDV/SOF was started (day 1, May 18, 20XX). On day 8 of dosing (May 25, 20XX), a fever of 38–39 °C and coughing were observed and then shortness of breath gradually appeared. There was no improvement in her condition, so the patient visited her primary care physician on day 16 of administration (June 2, 20XX). A chest roentgenogram showed bilateral shadows of ground glass in the lower lung fields, so the patient was brought urgently to the hospital on the same day. On the day of emergency transportation, blood tests, chest roentgenogram, computed-tomography scan and urine antigen tests were conducted at our hospital. Test results showed abnormal white blood cell count (10,100/μL), lactate dehydrogenase (941 U/L), platelets (57,000/μL), and C-reactive protein (CRP; 4.2 mg/dL) values, but sialylated carbohydrate antigen Krebs von den Lungen-6 (KL-6) value (303 U/mL) was within the normal value range. Chest roentgenogram demonstrated bilateral ground glass opacity (Fig. 2) in the lower lung fields, and chest computed tomography (CT) scans demonstrated significant ground-glass shadows in a mosaic pattern in the lower lobes of both lungs (Fig. 3). Because the condition was acute in onset and CT imaging indicated interlobular septal thickening, which was relatively thick, causes other than infectious disease were considered. Therefore, bronchoalveolar lavage (BAL) was urgently performed. No significant increase in the number of lymphocytes was detected, and the predominant cell differential was almost normal in the BAL fluid. Because the possibility of infection was low based on the results of imaging and BAL examinations, a drug-induced lung disease was suspected, LDV/SOF administration was discontinued, and steroid administration was started. Steroid pulse therapy with methylprednisolone sodium succinate was performed for 3 days starting the day after hospitalization (day 17, June 3, 20XX). After changing to prednisolone tablets for oral administration, the dose was gradually tapered. Blood and sputum cultures from the day of hospitalization were tested to rule out respiratory infection, and prophylactic azithromycin infusion was given until the results were known. An infectious disease was ruled out when the results of the blood and sputum culture showed no viable microbes and administration of azithromycin was discontinued after it had been administered for 5 days. On day 19 (June 5, 20XX), which was the fourth day after admission to our hospital, the patient coughed when taking deep breaths, but her dyspnea was improving. Because of the development of oral candidiasis, FLCZ 100 mg/day for 5 days was administered. On day 20 (June 6, 20XX), the respiratory condition improved and the auscultation findings tended to improve. On day 23 (June 9, 20XX), the white blood cell count, platelet count, and inflammatory marker levels improved. On day 27 (June 13, 20XX), the patient’s symptoms improved and she was discharged. During outpatient care on day 36 (June 22, 20XX), the cough remained but the shadow had almost disappeared on the chest roentgenogram (Fig. 2). On day 46 (July 2, 20XX), 30 days of steroid administration was completed. There was no deterioration in subjective symptoms and imaging results after completion of steroid administration. A drug-induced lymphocyte stimulation test (DLST) was performed on day 64 (July 20, 20XX), but the result was negative.

## Discussion and conclusions

LDV/SOF is one of the DAAs that inhibit enzymes translated from the NS3/4, NS5A, and NS5B regions. Interstitial lung disease is not indicated in the adverse effect information in the package insert for LDV/SOF, but four cases of interstitial lung disease were reported in the early post-marketing phase vigilance of the product [8], although the details of these cases are unknown. The Japanese Adverse Drug Event Report database includes 14 reports of interstitial lung disease caused by LDV/SOF from 2015 to 2019.

The mechanisms for the development of drug-induced lung diseases are almost unknown, but they are basically classified into the two following mechanisms: cytotoxic toxicity and allergic reaction (activation of immune system cells) [9–11]. Drugs causing lung disease because of cytotoxic toxicity include anticancer drugs and antiarrhythmic drugs. The anticancer agent enters the lung along with the blood flow and causes direct cell damage by contact with the endothelial cells of the pulmonary capillaries and the alveolar epithelial cells [11]. The allergic mechanism of drug-induced lung disease occurs with Chinese herbal medicines, antibacterial drugs, rheumatoid arthritis drugs, IFN, granulocyte colony-stimulating factor, etc. Antigenicity in the allergic mechanism is acquired when the drug or its metabolite binds as a hapten to lung tissue or to proteins and polysaccharides in the blood [11]. Since LDV/SOF is a weakly cytotoxic antiviral drug, we considered that the mechanism of drug-induced lung disease in this case was caused by the allergic mechanism. The onset time of drug-induced lung diseases is a few minutes to several years after administration. In general, the onset time is usually 2–3 weeks to 2–3 months after administration [9].

The patient’s medication history in this case showed no drugs taken before or after administration of LDV/SOF until the adverse reaction occurred, and there were no supplements or dietary supplements taken. Therefore, LDV/SOF is proposed as the cause of the suspected adverse effect. The symptoms of fever and cough that appeared to be the initial symptoms of the lung disorder appeared on day 8 (May 25, 20XX) after the start of LDV/SOF administration, and the patient was transported to the emergency department about one week later (day 16, June 2, 20XX). Laboratory examinations at our hospital showed an increase in white blood cell count, lactate dehydrogenase, and C-reactive protein, but KL-6 did not show an increase. KL-6 has been used as a biomarker for interstitial pneumonia and has been reported to be increased in drug-induced lung disease. However, it has also been reported that it may not increase because of the pathological condition of lung injury [9]. To the best of our knowledge, no pathological condition of lung disease due to LDV/SOF has been reported to date. It is considered that the clinical types of drug-induced lung diseases differ according to drug types. Therefore, we need to accumulate and consider more detailed reports.

Infections were ruled out by diagnostic imaging, BAL, blood culture, and sputum examination. Because drug-induced lung disease was suspected, LDV/SOF administration was immediately discontinued, and the symptoms of lung injury improved with steroid administration. Steroid therapy was changed to the oral route after 3 days of pulse administration when the respiratory symptoms improved, and was ended after about 1 month. Steroid treatment has been shown to be successful in treating allergic drug-induced lung diseases [9, 11, 12]. Since this case also showed a rapid improvement in laboratory values after steroid administration, it is considered to be an allergic drug-induced lung disease.

However, the result of a DLST performed two weeks after the steroid treatment was completed was negative. But the rate of a positive DLST test in drug-induced lung diseases is reported to be 66.9%, and even if the result was negative, it cannot be denied that this was a side effect of LDV/SOF.

When starting of administration LDV/SOF, treatment for rheumatoid arthritis in the patient had been already completed. While diagnosing interstitial lung disease, it caused by rheumatoid arthritis was ruled out because of no rheumatic symptoms and the shadow pattern of the lung CT image, and the test result for the anti-cyclic citrullinated peptide antibody was negative. Therefore, it is unlikely that interstitial lung disease developed because of rheumatoid arthritis. Hence, we considered that interstitial lung disease was caused drug-induced lung disease due to LDV/SOF. The diagnosis criteria for drug-induced lung disease caused by hypersensitivity reactions [9] were as follows: (1) Lung disorder observed after starting drug administration (1–6 weeks). (2) Initial symptoms include fever, cough, dyspnea, and rash (positive for 2 or more items). (3) Peripheral blood images show eosinophilia or leukocytosis. (4) Drug sensitivity test is positive. (5) Reproduction of pulmonary injury is observed by accidental re-administration. The definitive diagnosis satisfies items (1) and (4) or (5). The suspicion satisfies (1) and (2) or (1) and (3). In this case, the only medication administered when the lung injury occurred was LDV/SOF, which is considered a suspected drug because it meets the criteria for “suspect” in the above-mentioned diagnostic criteria.

This case suggests that there is a possibility of interstitial lung disease developing in patients undergoing hepatitis C treatment. Pharmacists should try to collect adverse effect reports for early identification of adverse effects. Pharmaceutical companies should also provide adverse effect information as soon as possible.

## Data Availability

Data used in this case report will not be shared due to the risk of identifying an individual, although patient’s data are presented in the main paper. The Japanese adverse drug event report database provided by PMDA can be accessed it directly from https://www.pmda.go.jp/safety/info-services/drugs/adr-info/0004.html.

## Abbreviations

BAL: Bronchoalveolar lavage
CT: Computed tomography
DAAs: Direct acting antiviral agents
DLST: Drug-induced lymphocyte stimulation test
HCV: Hepatitis C virus
IFN: Interferon
KL-6: Sialylated carbohydrate antigen Krebs von den Lungen-6
LDV/SOF: Ledipasvir Acetonate/Sofosbuvir
